# Temozolomide-Induced Myelodysplasia

**DOI:** 10.1155/2010/760402

**Published:** 2010-03-04

**Authors:** Ethan A. Natelson, David Pyatt

**Affiliations:** ^1^Department of Internal Medicine, The Methodist Hospital, Smith Tower 1001, 6550 Fannin Street, Houston, TX 77030, USA; ^2^School of Public Health, University of Colorado, Summit Toxicology, L.L.P., 1944 Cedaridge Circle, Superior, CO 80027, USA

## Abstract

A patient who had received temozolomide (TMZ) as a single agent in treatment of malignant glioma developed therapy-induced myelodysplasia (T-MDS). TMZ is an orally active imidazotetrazine which methylates guanine residues in DNA, ultimately causing single and double-strand DNA breaks leading to apoptotic cell death. TMZ does not chemically cross-link DNA and is considered a nonclassical alkylating agent, similar in structure and activity to dacarbazine. Observations on this patient, and on similarly treated others, suggest that the cumulative dose threshold (CDT) for TMZ that predisposes to T-MDS and which may potentially lead to acute myeloid leukemia (T-AML) is around 18000 to 20000 mg/sq m. Although the incidence of T-MDS and the predisposing CDT of TMZ may differ from that of other potentially leukemogenic compounds currently and formerly used as chemotherapeutic agents, all alkylating agents, including TMZ, should be considered potentially leukemogenic when administered long term.

## 1. Introduction

Most instances of myelodysplasia (MDS) and acute myeloid leukemia (AML) are de novo or primary illnesses, and occur without known exogenous or environmental cause and at an increasing incidence with advancing age [1–3]. Failure of DNA repair mechanisms and the physiologic requirement for programmed DNA fracture and chromosome reassembly during normal hematopoiesis are possible endogenous causes [1]. 

*Secondary *forms of MDS/AML are less common and may occur in the setting of pre-existing hematological or systemic disorders, after exposure to environmental toxins, or consequent to treatment of malignancy or circumstances requiring prolonged immunosuppression [4–7]. More and more cases of event-related or therapy-induced MDS/AML are being recognized. Recent studies suggest that individuals with this subset of *secondary* MDS/AML may have particular predisposing genetic loci [8, 9]. It is generally believed that *secondary* forms of MDS/AML are more refractory to therapy than are de novo illnesses, and as recently reported, even with equivalent low-risk chromosome aberrations [10–12]. The bone marrow histopathology of T-MDS more often shows multilineage dysplasia, fibrosis and hypoplasia than is observed in de novo forms of MDS making comparable classification difficult along guidelines designed for de novo MDS [12, 13]. This seems particularly true of examples of *secondary* MDS consequent to excessive occupational exposures to benzene, an obsolete chemotherapy drug [2, 14–17].

Alkylating agent-induced MDS/AML was first described after treatment of multiple myeloma with melphalan [18]. Many subsequent observations suggested that MDS/AML after therapy with alkylating agents was not idiosyncratic, but directly related to their cumulative dose, regardless of mode of administration [19]. The clinical features of such *secondary* AML syndromes include a MDS phase in as many as 70% of cases [2, 4, 5]. By contrast, de novo AML typically begins abruptly without a period of MDS. There also appears to be a latency period and a window of opportunity, from exposure to onset of hematologic neoplasm, generally described as between 2 and 10 years after a potentially leukemogenic CDT is reached [5]. Certain cytogenetic aberrations in bone marrow cells are also more likely to occur after exposure to alkylating agents. Such aberrations involve variable losses of genetic material from chromosomes 5 and 7 in as many as 50–70% of cases [2–5, 10]. Moreover, some alkylating agents may be far more leukemogenic than others, and the CDT for each compound varies considerably [6, 7, 19, 20]. Expanded use of a newer alkylating agent, TMZ, developed to facilitate central nervous system drug penetration in patients with cerebral neoplasms, has become a standard of care [21, 22]. Unfortunately, its mechanism of action suggests major efficacy only for central nervous system tumors bearing specific genetic markers [23, 24]. The increasing reports of MDS/AML syndromes following the use of TMZ serve as a reminder that alkylating agents cannot be safely used continuously and long-term unless the intent is palliation, rather than cure.

## 2. Case Report

A 64-year-old previously healthy right-handed man experienced the sudden onset of grand mal seizures. Cranial CT scans demonstrated a small left temporal lobe lesion. Microscopic examination after its excision showed histology consistent with astrocytoma, and clear tumor margins. He received postoperative radiation therapy, along with small oral doses of TMZ as a radiosensitizer. This drug was then continued at 200 mg/sq m/day, 5 days each month, for 18 courses (total dose, approximately 20,000 mg/sq m or 40,000 mg). Pretreatment blood counts gave entirely normal values. Transient leukopenia and thrombocytopenia occurred during chemotherapy, but the patient did not require use of hematological growth promoting factors or blood component transfusion. His blood counts returned to normal, but several months after completion of all therapy, a mild pancytopenia became evident, and gradually worsened. Three years after treatment, the peripheral blood film demonstrated macrocytosis and typical bilobed Pelger-Huet granulocytes [25]. The bone marrow examination at that time showed normal total cellularity but demonstrated 3% blast forms and trilineage dysplasia. Conventional cytogenetics and FISH (*fluorescent in situ hybridization*) probes confirmed isolated monosomy 7 in 80% of the marrow metaphases and no alterations in chromosome 5. A diagnosis of high-risk MDS was evident. Allogeneic bone marrow transplantation was contemplated, but 5 months after his diagnosis of MDS, recurrence of the brain tumor was identified in the left frontal lobe behind the optic nerve. Re-biopsy confirmed astrocytoma, and he received additional radiation therapy along with a course of azacitidine for his MDS. This latter treatment resulted in a severe pancytopenia, but eight months later, his blood counts were considerably improved from those obtained at the time of his bone marrow examination, and he was essentially asymptomatic. He died from progression of the cerebral neoplasm 43 months following its initial diagnosis without conversion from MDS to AML.

## 3. Discussion

Kyle, in 1970, first called attention to the leukemogenic potential of the alkylating agent, melphalan, when used in the treatment of multiple myeloma [18]. The importance of a CDT among alkylating agents as a cause of *secondary* AML was soon appreciated with analysis of studies reporting the incidence of AML following increasing cycles of MOPP (mechlorethamine, vincristine, procarbazine, prednisone) chemotherapy for Hodgkin's lymphoma [19]. Greene emphasized the duration of treatment and the total dose administered as predictive of a greater leukemic risk following administration of cyclophosphamide, perhaps the alkylating agent most thoroughly studied in regard to *secondary* AML [26]. The potentially leukemogenic cumulative dose for cyclophosphamide has been estimated at 20,000 mg and is about ten-fold that seen with another well-studied alkylating agent, chlorambucil [19]. The alkylating agent/CDT concept in leukemogenesis has been reconfirmed in a more recent study demonstrating the appearance of, and abrupt rise in, the incidence of *secondary* AML in patients treated for breast cancer and receiving cyclophosphamide in doses above 8,000 mg/sq m along with other agents [6]. This increased risk of T-MDS/AML was not observed in patients treated with lower doses. For other types of chemotherapeutic agents that may cause *secondary* AML/MDS, such as topoisomerase II inhibitors, the mean latency is shorter, and different chromosome aberrations such as balanced translocations are common [4]. The existence of a CDT is probable but less well-defined since these agents are often used in combination chemotherapy. Additionally, a prodrome of MDS is far less common [2, 4, 5, 7, 11]. 

TMZ is a novel chemotherapy drug that is structurally related to dacarbazine. It has the advantage of a rapid absorption following oral administration with almost 100% bioavailability. Moreover, drug levels in the central nervous system are 30–40% of TMZ plasma concentrations [27, 28]. As such, this drug has gained much attention as front-line therapy for malignant gliomas, for which effective therapy remains poor. TMZ does not undergo significant liver metabolism and hydrolyzes rapidly to form the active alkylating metabolite in plasma. 

TMZ is considered a non-classical alkylating agent in that it does not cross-link DNA strands. Its metabolites methylate DNA, particularly on guanine sites. The formation of O^6^-methylguanine generates single and/or double strand DNA breaks when the affected cell line attempts to repair this alteration via mismatch repair mechanisms and apoptosis results. O^6^–methylguanine-DNA methyltransferase (MGMT) plays a critical role in protecting cells from alkylating agents that target O^6^-guanine, including TMZ [29, 30]. This enzyme transfers the inappropriate methyl group from the guanine to a cysteine residue which signals for its proteasome-mediated degradation destruction via an ubiquitination pathway [31]. Thus, TMZ mediated apoptosis appears to be especially effective in MGMT negative tumors or in tumors with a hypermethylated MGMT promoter [28, 31]. For this reason knowledge of the MGMT promoter status provides clinically useful information regarding the potential efficacy to TMZ and other alkylating agents in a variety of tumor systems [30]. Additional studies report that TMZ also induces cell cycle arrest at the G2-M checkpoint and /or disrupts tubulin formation [32, 33].

Lacking ability to cross-link DNA, TMZ has less direct toxicity to hematopoietic progenitor cells than classical alkylating agents. As a result, myelosuppression is relatively mild and not often dose-limiting. However, scattered reports describe prolonged myelosuppression and even aplastic anemia, in addition to the delayed syndromes of T-MDS/AML following therapy with TMZ [34–36]. Hypermethylation-induced gene silencing is a feature of MDS and *secondary* AML, in comparison with de novo AML and may have something to do with the lack of response to standard chemotherapy in these syndromes and could also favor the induction of T-MDS/AML [37]. 

Currently, TMZ is used in treatment of various types of gliomas, typically in concert with radiation therapy and as a radiosensitizing agent [38]. In more recent reports, it has been combined with other agents with different mechanisms of action in an attempt to find a more potent treatment advantage over radiation alone following debulking neurosurgery [39, 40]. TMZ has also been studied as a chemotherapeutic agent in melanoma and other solid tumors and in hematological disorders but little clinical data as yet exists.

 The patient reported here received TMZ at a total dose of 20,000 mg/sq m prior to the diagnosis of T-MDS. In another instance where TMZ was used primarily as a single agent, the total dose administered before onset of T-MDS was quite similar, at 18,750 mg/sq m [41]. In a series of 7 patients with gliomas, all of whom received TMZ and who later developed alkylating agent-induced MDS, 2 were treated with TMZ alone at doses of 24,000 and 28,000 mg/sq m prior to T-MDS/AML. The median dose of TMZ in this cohort was 18,000 mg/sq m, but 5 of the 7 patients had also received substantial therapy with other chemotherapeutic agents [21]. In the patient reported here, and in the 3 additional patients previously reported who developed T-MDS following TMZ alone, MDS was confirmed 28 to 36 months following treatment. Additional occurrences of T-MDS/AML in patients receiving TMZ in the treatment of gliomas have been described, but the use of multiple chemotherapy drugs in these patients prevents any conclusions about the contributions of individual compounds [42, 43].

Benzene is an obsolete chemotherapeutic agent briefly used in the early approach to treatment of chronic myeloid leukemia [1]. It continues to be of major scientific interest because it is a well-known potential cause of *secondary* MDS/AML via occupational exposures at high cumulative dose levels [1, 2, 44–47]. Its mechanism of action is unknown, but it has been termed a radiomimetic compound because of the similarity in the myeloid cell chromosome aberrations it may produce to those identified after both therapeutic radiation exposure and following treatment with alkylating agents. Some of the metabolites of benzene also exhibit anti-topoisomerase II activity, the importance of which in terms of its mechanism of leukemogenesis is debated [44–46]. Regulatory agencies, epidemiological studies and peer-reviewed medical literature typically cite cumulative benzene exposures in excess of 40 ppm years as the minimum exposure required to potentially cause *secondary* MDS/AML [2, 47, 48]. However, exposure levels well in excess of these values may not result in a statistically significant excess incidence of AML [49, 50].

In order to compare potentially leukemogenic doses of benzene with those of other chemicals, the typical exposure metric of ppm-years must be converted to the more clinically relevant mg/sq m. An estimate of the mg absorbed dose of benzene given a constant 1 ppm (32 mg/m^3^ = 1 ppm) inhalation over an 8 hour daily work schedule during a 250 day work year and continuing over a 40 year period employing standard measurements for respiratory rate, tidal volume and absorbed dose, would yield a value around 92,000 mg. Assuming the worker measured 2 sq m in body surface area, this would suggest that the minimum CDT necessary to predispose to secondary MDS/AML would be about 46,000 mg/sq m. With controls on the amount of benzene allowable as a trace contaminant in modern solvents, it has become more difficult to achieve the cumulative dose levels required to initiate leukemogenesis in workers in developed countries [51]. The wide range of the estimated CDT necessary to predispose to *secondary* MDS/AML for selected and well-studied modern chemotherapeutic agents including TMZ and in comparison with benzene is shown in Table 1 [6, 7, 18–20, 26, 47]. 

Most individuals receiving exceptionally large doses of alkylating agents over an extended period do not develop T-MDS/AML. This also is true in patients receiving TMZ [22]. However, considering the limited survival of those with malignant gliomas, in comparison with the wide range of latency in alkylating agent-induced MDS/AML, it is understandable why more examples of this hematological complication have not been described. This relatively short survival interval also makes it more difficult to compare the incidence of leukemogenesis from TMZ with that caused by alkylating agents commonly used in the treatment of neoplasms which are associated with a long survival, such as Hodgkin's lymphoma, testicular cancer and breast cancer. Nevertheless, patients and their physicians should be aware that prolonged use of TMZ as a single agent is not innocuous and carries the potential to cause T-MDS/AML. The CDT that predisposes to these hematological syndromes estimated on limited case material appears to be around 18,000 to 20,000 mg/sq m. Based on the extensive medical literature on the subject, any alkylating agent in long-term administration must always be considered potentially leukemogenic. 

## Figures and Tables

**Table 1:** Estimated CDT for selected potentially leukemogenic agents.

Compound	Activity	CDT (mg/sq m)	Latency to MDS/AML (yrs)
Temozolamide	alkylating agent	18–20,000	0.75–3
Cyclophosphamide	alkylating agent	8–10,000	2–10
Alkeran	alkylating agent	80	2–10
Mechlorethamine	alkylating agent	60	2–10
Etoposide	topo II inhibitor	2,000	1–4
Mitoxantrone	topo II inhibitor	60	0.50–5
Benzene	unknown	46,000	2–10
